# Are Wednesday's Children Full of Woe? Children's Differences in Personality Are Independent of Day of Birth

**DOI:** 10.1111/jopy.70005

**Published:** 2025-07-11

**Authors:** Emily Wood, Anna Brown, Kirsty Wilding, Florence A. R. Oxley, Helen L. Fisher, Louise Arseneault, Avshalom Caspi, Terrie E. Moffitt, Sophie von Stumm

**Affiliations:** ^1^ Department of Education University of York York UK; ^2^ University of Northampton Northampton UK; ^3^ King's College London Social, Genetic & Developmental Psychiatry Centre, Institute of Psychiatry, Psychology & Neuroscience London UK; ^4^ ESRC Centre for Society and Mental Health, King's College London London UK; ^5^ Department of Psychology and Neuroscience Duke University Durham North Carolina USA; ^6^ Department of Psychiatry and Behavioral Sciences Duke University Durham North Carolina USA; ^7^ PROMENTA, Department of Psychology University of Oslo Oslo Norway

**Keywords:** child development, individual differences, longitudinal research

## Abstract

**Introduction:**

Nursery rhymes, which are rich in literary devices, benefit children's language learning. Less is known about the influence that nursery rhymes' messages may have on children's development. We focused on “Monday's Child,” a popular nursery rhyme that alleges children's day of the week of birth forecasts their differences in personality and physical traits.

**Methods:**

Data came from E‐Risk, a UK population representative, longitudinal cohort study of 2232 same‐sex twin children (with 93% retention). We used linear regression models to test whether the day of the week of birth predicted personality and physical traits at ages 5–18 years.

**Results:**

Being born on Monday through Saturday did not predict children's personality and physical traits as implied by the “Monday's Child” rhyme. Being born on Sunday was also not associated with children's traits across measures. These results were unchanged after covariate adjustment (i.e., children's sex, birthweight, and socioeconomic status).

**Conclusion:**

We showed that children's differences in personality and physical traits are independent of their day of the week of birth. These findings suggest that nursery rhymes' messages are unlikely to influence children's development, at least those conveyed by “Monday's Child”.

## 1 Introduction

Monday's child is fair of face,
Tuesday's child is full of grace,
Wednesday's child is full of woe,
Thursday's child has far to go,
Friday's child is loving and giving,
Saturday's child works hard for his living,
And the child that is born on Sunday
Is fair and wise and good and gay.

Engaging in nursery rhymes may aid children's language development, literacy skills, and cognitive abilities (Bryant et al. 1989; Dunst et al. 2011; Starr, Oginni, and von Stumm 2024), because nursery rhymes are rich in alliteration, vocabulary, onomatopoeia, and imaginative imagery (Bryant et al. 1989; Kenney 2005). However, whether the messages that nursery rhymes convey also affect children's personality development has not been previously studied. Messages that are transmitted via other media, such as television, film, books, and merchandize, have been shown to influence children's attitudes and behaviors (e.g., Coyne et al. 2016, 2021; Kirkorian et al. 2008). For example, engaging with Disney princess media and products during early childhood was associated with an increase in female gender‐stereotypical behavior (Coyne et al. 2016). Yet, Disney princess culture changed over the past decade, with films that feature androgenous princesses and narratives of female empowerment. More recently, identifying with Disney princesses and playing with Disney princess toys during the preschool years were associated with developing positive body images and egalitarian attitudes in early adolescence (Coyne et al. 2021). Here, we addressed whether messages from nursery rhymes may also predict children's personality development, akin to the influences of Disney princess media on attitudes. We focused on “Monday's Child,” a popular British nursery rhyme which implies that the day of the week on which a child is born forecasts their personality and physical traits.

### 1.1 “Monday's Child” in Popular Culture

First recorded in 1833 (Bray 1838), the “Monday's Child” nursery rhyme continues to be known by British people of all ages, and its messages are omnipresent in popular culture. For example, in 2022, Netflix released the hit series “Wednesday,” whose protagonist, Wednesday Addams, is emotionally reserved and morbid, with a dark, sarcastic personality. The first episode of the series was even titled “Wednesday's child is full of woe”. The show crossed one billion viewing hours within 28 days of its release and debuted at number one on Netflix in 83 countries (Oganesyan 2022). But Wednesday Addams' imprint on popular culture predates Netflix: The cartoonist Charles Addams created Wednesday as a member of the fictional Addams family, first aired on ABC in 1964, who delights in the macabre and ignores social convention (Miserocchi 2010). Addams named Wednesday after the “Monday's Child” nursery rhyme in reference to her “woeful” character. Later, in 2001, ABC also aired “Snow White: The Fairest of Them All,” featuring seven dwarfs who were named after the days of the week and had personalities to match, in line with the “Monday's Child” nursery rhyme (Thompson 2002). Other examples of the perpetuation of “Monday's Child” in popular culture include Will Young's 2004 album “Friday's Child,” whose single of the same title featured the lyrics: “Monday's got a beautiful baby/And Wednesday's child can never win/Little Saturday will work till he's crazy/But Friday's child was born to give”. The album sold over 1.8 million copies worldwide and went five times platinum in the United Kingdom (Kutner 2022). In 1999, David Bowie reflected on his life, loves, and career in the song “Thursday's Child”—although Bowie himself was born on a Wednesday, he identified with the long and onerous journey to happiness and success that is predicted for Thursday's children (Draper 2024). The enduring presence of the “Monday's Child” nursery rhyme in popular culture suggests that children and parents across generations have been exposed to the rhyme and thus, may have been influenced by its messages.

### 1.2 How the Messages of “Mondays Child” May Influence Children's Personality and Physical Traits

There are several ways in which messages of the “Monday's Child” nursery rhyme may influence children. One is known as confirmation bias, where people seek evidence which confirms their existing beliefs (Rabin and Schrag 1999; Remmerswaal et al. 2014). For instance, children found that the events of a recent day more closely matched the prediction for their particular astrological sign when the astrological signs were presented along with the predictions than when they were not (Munro and Munro 2000). In the same way, children may perceive their everyday behaviors to match the predictions of the Monday's Child nursery rhyme, and thus, accept the rhyme's verses as true. For example, children born on Wednesdays may attribute their feelings of melancholy, worry, or misfortune, which all children experience on occasion, to their day of birth. As a result, they may perceive themselves to experience these emotions more often or intensely than do children born on a different day.

Another way in which “Monday's Child” nursery rhyme may influence children is the self‐fulfilling prophecy, where people's expectations about their situation cause them to become true (Madon et al. 2011). For example in education settings, children who have greater academic self‐belief tend to perform better in school than those who are less convinced of their academic acumen (Valentine et al. 2004). If children believe themselves to possess the trait assigned to them by “Monday's Child,” they may develop that trait as a result. For example, children born on Mondays may think of themselves as facially more attractive than children born on other days. Thus, they may develop greater confidence and self‐esteem (Diener et al. 1995), which in turn could make them appear more good‐looking to others (Zeigler‐Hill and Myers 2011).

A third pathway of influence is through parents and other caregivers who internalized the messages that are conveyed in “Monday's Child”. For instance, parents may raise their children—perhaps inadvertently—according to the rhyme's predictions (cf. Pinquart and Ebeling 2020). For example, parents of Tuesday‐born children may enroll their children in a ballet class as they foresee them being “full of grace” or invest more in their Thursday‐born children's education as they expect them to have “far to go”. Moreover, parents' perception of their children may influence children's self‐image—a phenomenon known as the Looking Glass Self Hypothesis (Silva et al. 2021). The looking‐glass self refers to when individuals base their sense of self on how they understand that others view them. For instance, Tuesday‐born children may believe that their parents view them as being “full of grace” and consequently develop a self‐image that builds on “grace”. These emphases in parenting may shape children's development of personality and physical traits according to the predictions of the “Monday's Child” nursery rhyme.

### 1.3 Day of Birth as a Predictor of Personality Differences

Previous studies have reported that beliefs about the significance of day or time of birth can manifest in children's personality differences. For example, the Asante people, who are native to modern‐day Ghana, believe that boys born on Mondays are quiet and peaceful in character, whereas boys born on Wednesdays are quick‐tempered and aggressive (no significance is attributed to girls' day of birth; Jahoda 1954). Analyses of the region's Juvenile Court records showed that delinquent boys were less often born on Mondays than on other days of the week. Furthermore, boys born on Wednesdays were more often charged with committing “offences against the person,” such as assault or fighting, compared with boys born on any other day (Jahoda 1954). These findings imply that the Asante people's beliefs about the effect of children's day of birth on their personality can indeed translate into observable differences in children's behaviors.

Another study tested if children born in the Year of the Dragon according to the Chinese zodiac calendar (e.g., 2012, 2024, 2036) are destined for greatness and good fortune as compared to children born in years of other, less auspicious zodiac signs (Mocan and Yu 2020). In nationally representative survey data from China (*N* ~ 3000 to 13,000), children born in the Year of the Dragon were more likely to achieve higher scores in middle school and university entrance exams and to earn Bachelor degrees than children born in other years (Mocan and Yu 2020). This association could be attributed to differential parenting, because Chinese parents invest more in the education of children who are born in “lucky” years, such as the Dragon year, compared to those born in “unlucky” years (Tan et al. 2024). These findings suggest that parents' beliefs about the auspiciousness of their children's year of birth influence their parenting behaviors and by extension, their children's developmental outcomes.

The findings from Jahoda (1954) and Mocan and Yu (2020) offer partial empirical support for the three theoretical pathways specified above, including confirmation bias, self‐fulfilling prophecy, and the looking glass self. That is, children and parents may seek—whether consciously or not—confirmation of the nursery rhymes' predictions in their children's behavior and characteristics (i.e., confirmation bias), as implied by both earlier studies (Jahoda 1954; Mocan and Yu 2020). Likewise, children may behave in line with the predictions of the nursery rhyme to meet the expectations that they perceive their parents and others to hold for them. The theories of the self‐fulfilling prophecy and looking glass self hypothesis seem particularly relevant in the context of Mocan and Yu's (2020) study that demonstrated how the parenting of children born in the Year of the Dragon explained their advanced academic performance.

Other research has rejected the hypothesis that birth dates are associated with developmental differences or people's outcomes. For example, data from the UK Labor Force Survey found no link between being born on Friday 13th, a date that is widely believed to bring bad luck and misfortune (Rauch 2021), and the likelihood of experiencing unemployment, earning low wages, or being unmarried than being born on other dates (Fidrmuc and Tena 2014). Similarly, the rate of hospital admissions on Friday 13th, on days with a full moon (associated in folklore with insomnia and insanity, i.e., lunacy), or on Friday 13th with a full moon did not differ from that on other days in data from Switzerland (Exadaktylos et al. 2001). Finally, the mortality of patients suffering acute coronary syndrome (e.g., a heart attack) was no higher on Friday 13th than on other days according to a South Wales hospital registry (Protty et al. 2016). These findings question whether prevalent beliefs about dates can exert meaningful influence on behavior.

### 1.4 The Current Study

We used data from E‐Risk (https://www.eriskstudy.com), a UK population‐representative study of families who had twin children born in 1994 to 1995 in England and Wales, to test whether day of the week of birth relates to personality and physical traits, as predicted by the “Monday's Child” nursery rhyme. E‐Risk is a longitudinal cohort study that assessed a wide range of personality and physical traits when the study children were 5, 7, 10, 12, and 18 years old. From the E‐Risk data dictionary, we identified measures that captured the personality and physical traits predicted by the “Monday's Child” nursery rhyme. For example, we equated “fair of face” with being perceived as more attractive or good‐looking, which we inferred from independent observer ratings based on video recordings or in‐person interactions at ages 5, 10, 12, and 18 years. For all personality and physical traits predicted by the “Monday's Child” nursery rhyme, repeated multi‐measure, multi‐informant assessments were available from E‐Risk.

The exact wording and interpretations of the meaning of “Monday's Child” nursery rhyme's predictions varied across time and contexts (Fox 2000; Herbst 2023). For example, “Tuesday's child is full of grace” could be interpreted in the way that children born on Tuesdays are gracious in personality, meaning courteous, kind, and pleasant. Yet, the line could also be understood to refer to grace in physical mobility, such as being nimble and light on one's feet. Similarly, we might infer from “Thursday's child has far to go” that children born on Thursdays are disadvantaged in life, so they have “further” to go and overcome more obstacles than others to achieve success. Alternatively, and perhaps more commonly, the line “has far to go” is associated with having good prospects for achieving success, where going “further” implies outperforming others. Where possible, we accommodated these different interpretations of the “Monday's Child” nursery rhyme in our analyses. For example, we included both measures of gracious personality and of physical mobility to assess “full of grace”. That said, we acknowledge that our study covered only some, but not all the possible interpretations of the “Monday's Child” nursery rhyme.

## 2 Materials and Methods

### 2.1 Sample

The Environmental Risk (E‐Risk) Longitudinal Twin Study is a longitudinal cohort study that was constructed in 1999–2000, when 1116 families (90% White) from England and Wales with same‐sex 5‐year‐old twins (*n* = 2232, born in 1994–1995) participated in a home‐visit assessment (Moffitt and E‐Risk Study Team 2002). E‐Risk families are representative of other UK families in the 1990s (Odgers et al. 2012). The sample is representative of all socioeconomic conditions in Britain, with E‐Risk families' distribution on ACORN and the UK Index of Multiple Deprivation (i.e., neighborhood‐level socioeconomic indexes) closely matching those of households nationwide (Odgers et al. 2012). The total sample comprised 56% monozygotic (MZ = 621) and 44% dizygotic (DZ = 495) twin pairs, with sex evenly distributed within zygosity (49% male). Follow‐up home visits were conducted when twins were aged 7 (98% retention rate), 10 (96%), 12 (96%), and 18 years (93%). Home visits at ages 5, 7, 10, and 12 years old included assessments of the twins as well as of their mothers or other primary caregivers. With parental permission, questionnaires were also posted to the twin's teachers, with 94% being returned at age 5, 91% at age 7, 86% at age 10, and 80% at age 12. At age 18, each twin of a pair was assessed by a different interviewer.

The E‐Risk Study operate a managed access process to protect the privacy of their participants and the integrity of their study. The data used for this research is available upon request to the E‐Risk Study steering committee (https://www.eriskstudy.com/data‐access/).

### 2.2 Measures

Table 1 gives an overview of all measures across assessment ages.

**TABLE 1:** Description of measures of the nursery rhyme's predictions.

Rhyme	Personality trait	Measures	*N*_items_
Monday's child is *fair of face*	Attractiveness	Attractiveness ratings at ages 5, 10, 12, and 18	4
Tuesday's child is *full of grace*	Physical fitness^a^	PE (physical education) grades at ages 7, 10, and 12	3
Wednesday's child is *full of woe*	Withdrawn, anxious, depressed	Child Behavior Checklist at ages 5, 7, 10, and 12	4
Thursday's child *has far to go*	Educational achievement	Secondary school qualifications at age 18	1
Friday's child is *loving and giving*	Prosocial behavior	Revised Rutter Parent Scale for School‐Age Children at ages 5, 7, 10, and 12	4
Saturday's child *works hard for a living*	Hardworking behavior	Achenbach Teacher Report Form & Strengths and Difficulties Questionnaire at ages 5, 7, 10, 12, and 18	9
The child that is born on Sunday is *fair and wise and good and gay*	All of the above		

^a^ For an alternative interpretation, including being well‐poised, gracious, and confident, see the Supporting Information.

#### 2.2.1 Day of Birth

Day of birth was recorded from Monday through Sunday, based on the twins' dates of birth which were reported by mothers when their twin children were 18 months old. There were nine instances of twin siblings being born on separate days in our dataset.

#### 2.2.2 Fair of Face

We equated “fair of face” with being perceived as more attractive or good‐looking. At age 5 years, three independent coders, who did not conduct the home interviews for directly assessing the twins, rated twins' attractiveness using video recordings on a 7‐point Likert scale (1 = extremely unattractive, 2 = very unattractive, 3 = unattractive, 4 = somewhat attractive, 5 = attractive, 6 = very attractive, 7 = extremely attractive). Inter‐rater reliability was 0.61 and the three raters' scores were averaged to produce one combined score. At ages 10 and 12 years, twins' attractiveness was rated by a single rater after an in‐person interaction, using the same 7‐point Likert scale as before. At age 18, an interviewer rated an “unattractive” item after an in‐person interaction with each twin, using a 3‐point Likert scale (0 = no, 1 = a little/somewhat, 2 = yes), which we reverse‐coded. All measures of attractiveness were *z*‐transformed and averaged together to create one composite attractiveness score (*M* = −0.02, SD = 0.71, *α* = 0.61).

#### 2.2.3 Grace

We interpreted “full of grace” in terms of physical competences, fitness, and motor abilities, which are indexed by Physical Education (PE) grades in UK schools (*National Curriculum in England* n.d.). Teachers reported on twins' PE performance using a 5‐point Likert scale (1 = far below classmates, 2 = somewhat below, 3 = average, 4 = somewhat above, 5 = far above) at ages 7, 10, and 12 years. All PE scores were z‐transformed and averaged together to create a PE composite (*M* = 0.00, SD = 0.82, *α* = 0.64). In separate, additional analyses (Table S8), we tested an alternative interpretation of the term “full of grace,” using interviewers' ratings of how “well‐poised, gracious, and confident” each twin was at age 18 years on a 3‐point Likert scale (0 = no, 1 = a little/somewhat, 2 = yes).

#### 2.2.4 Woe

We assessed “full of woe” in terms of emotionality, including the tendency to be withdrawn, anxious, and depressed. Emotionality was assessed when the twins were aged 5, 7, 10, and 12 years using the Child Behavior Checklist in interviews with mothers and the Teacher Report Form by post for teachers (Achenbach 1991a, 1991b). The emotionality scale is the sum of items on the withdrawn and anxious/depressed subscales. The internal consistencies of mothers' and teachers' reports were 0.88 and 0.93, respectively. To create a cross‐informant emotionality composite, ‐mothers' and teachers' ratings were *z*‐transformed and averaged together (*M* = 0.00, SD = 0.81, *α* = 0.84).

#### 2.2.5 Far to Go

Having “far to go” could be interpreted as having a long and successful future ahead or as having a long way to go to success. We measured this in terms of educational achievement, because children who do well in school tend to have more favorable life outcomes (Starr, Haider, and von Stumm 2024). In E‐Risk, twins reported their educational qualifications at age 18 years on a 4‐point scale from “no qualification” to “GCSE grades D‐G” to “GCSE grades A*‐C” to “A‐level”. The educational qualifications were z‐transformed (*M* = 0.00, SD = 0.99).

#### 2.2.6 Loving and Giving

We equated “loving and giving” with displaying prosocial behaviors. Two items from the Revised Rutter Parent Scale for School‐Age Children (Sclare 1997)—“tries to stop quarrels or fights” and “kind to animals”—were rated on a 3‐point scale (0 = not true, 1 = somewhat or sometimes true, 2 = very true or often true) by mothers when twins were aged 5, 7, 10, and 12 years. All prosocial behavior scores were *z*‐transformed and averaged together to create a prosocial behavior composite (*M* = 0.02, SD = 0.80, α = 0.80).

#### 2.2.7 Works Hard for a Living

We likened “works hard for a living” to the child's motivation to work hard in school, as rated by teachers using the Achenbach Teacher Report Form (TRF; Achenbach 1991b) and the Strengths and Difficulties Questionnaire (SDQ; Goodman 1997). At ages 5 and 7, the item “compared to typical pupils of the same age how hard is she/he working?” was rated on a 7‐point Likert scale (1 = much less, 2 = somewhat less, 3 = slightly less, 4 = about average, 5 = slightly more, 6 = somewhat more, 7 = much more). At ages 10 and 12, three items including “how often do you act to curb disruptive behaviour?,” “how often do you give child extra encouragement to take part?,” and “how often do you act to keep child's attention in class?” were rated on a 7‐point Likert scale (as above) and reverse scored for this analysis. At age 18, the co‐twin or mother rated how well the statement being a “hard worker” fit the twin (0 = no doesn't apply, 1 = yes applies somewhat, or 2 = yes certainly applies). All hardworking measures were *z*‐transformed and averaged together to create a hardworking composite (*M* = 0.00, SD = 0.66, *α* = 0.82).

#### 2.2.8 Fair and Wise and Good and Gay

We interpreted “fair and wise and good and gay” as excelling on all measures of personality and physical traits described above, including being attractive, physically fit, experiencing fewer internalizing emotions, achieving higher educational qualifications, demonstrating more prosocial behaviors, and working harder in school.

#### 2.2.9 Sex and Birthweight

Mothers completed a milestone questionnaire at first contact when the twins were 18 months old. Twins' sex was recorded as binary (male or female). Twins' birthweight, recalled by mothers, was recorded in grams rather than as dichotomous classification of low birthweight (LBW) versus normal birthweight (NBW), because the conventional cutoff for NBW (> 2500 g) is based on singleton rather than twin births, who on average have lower gestational ages and birthweights. In E‐Risk, 51% of twins weighed less than 2500 g at birth and mean gestational age was 36 weeks (SD = 3, range 24–43 weeks).

#### 2.2.10 Socioeconomic Status

Family socioeconomic status (SES) was indexed by a standardized composite of parental income (i.e., total household income), education (i.e., highest parent qualification), and occupation (i.e., highest parent occupation) when the twins were 5 years old. The three SES indicators were highly correlated (*r* (2230) = 0.57–0.67, *p* < 0.05) and loaded significantly onto one latent factor (factor loadings = 0.80, 0.70, and 0.83 for income, education, and occupation, respectively). The population‐wide distribution of this latent factor was then divided into tertiles (1 = low SES, 2 = mid SES, 3 = high SES).

### 2.3 Statistical Analysis

Our analyses were preregistered (https://sites.duke.edu/moffittcaspiprojects/files/2024/05/Wood_2024_Day‐of‐birth‐and‐outcomes.pdf). All primary data and study materials are available upon request to the E‐Risk study steering committee (https://eriskstudy.com/data‐access/). The scripts used for the present analysis can be found here osf.io/qxv4z. We acknowledge that there are analogous methods of modeling the day of birth variable to ours. Other researchers may test alternative models building on these data and our scripts.

Individual items for our measures were *z*‐transformed and reverse‐coded where appropriate, before averaging them into composites. We estimated Cronbach's alpha for each composite (*α* = 0.61–0.84, see Table S1 for details). The continuous covariate, birthweight, was also *z*‐transformed prior to analysis. Binary variables were created to reflect being born on a specific day of the week (e.g., Monday), coded as 1, versus being born on any other day of the week, coded as 0.

We fitted linear regression models to test whether day of the week of birth was associated with children's personality and physical traits according to the “Monday's Child” nursery rhyme, using the lavaan package in R (Rosseel 2012). Because data were assumed to be missing at random (and participation rates were so high that E‐Risk has little missing data), Full Information Maximum Likelihood (FIML) estimation was applied (Cham et al. 2017). Standard errors were clustered at the family level to account for the nonindependence of observations (i.e., two twins per family).

In our linear regression models, we first entered the day of the week of birth as a predictor of the respective child outcome variable (e.g., the attractiveness composite was predicted by being born on Mondays versus any other day of the week). We then added our covariates including children's sex, birthweight, and SES. We evaluated beta weights with 95% confidence intervals (CI), *p*‐values, and model *R* ^2^ to interpret the results. Six models were built for births occurring on Mondays through Saturdays, respectively. For births on Sundays, we first fitted six linear models to test the binary predictor of being born on Sunday versus on another day of the week for each outcome. In additional (not preregistered) analyses, we tested if children born on Sundays had cumulatively more favorable personality and physical traits: we predicted composite scores across all measures after reverse‐coding the emotionality composite from the binary variable of being born on a Sunday versus on any other day of the week.

## 3 Results

Descriptive statistics of the study sample can be viewed in Tables S1 and S2. The frequency of twins' birth ranged from *N* = 105 on Sundays to *N* = 202 on Fridays (9.4% and 18.1% of E‐Risk's births, respectively; see Table S2 for details), in line with previous studies that showed higher birth rates on Fridays, presumably because inductions are more common before than during the weekend (Littleboy 2019). Our covariates, including children's sex, birthweight, and SES, did not vary significantly by day of birth (Figures S1–S3).

### 3.1 Is Day of the Week of Birth Associated With Children's Differences in Personality and Physical Traits?

**FIGURE 1:**
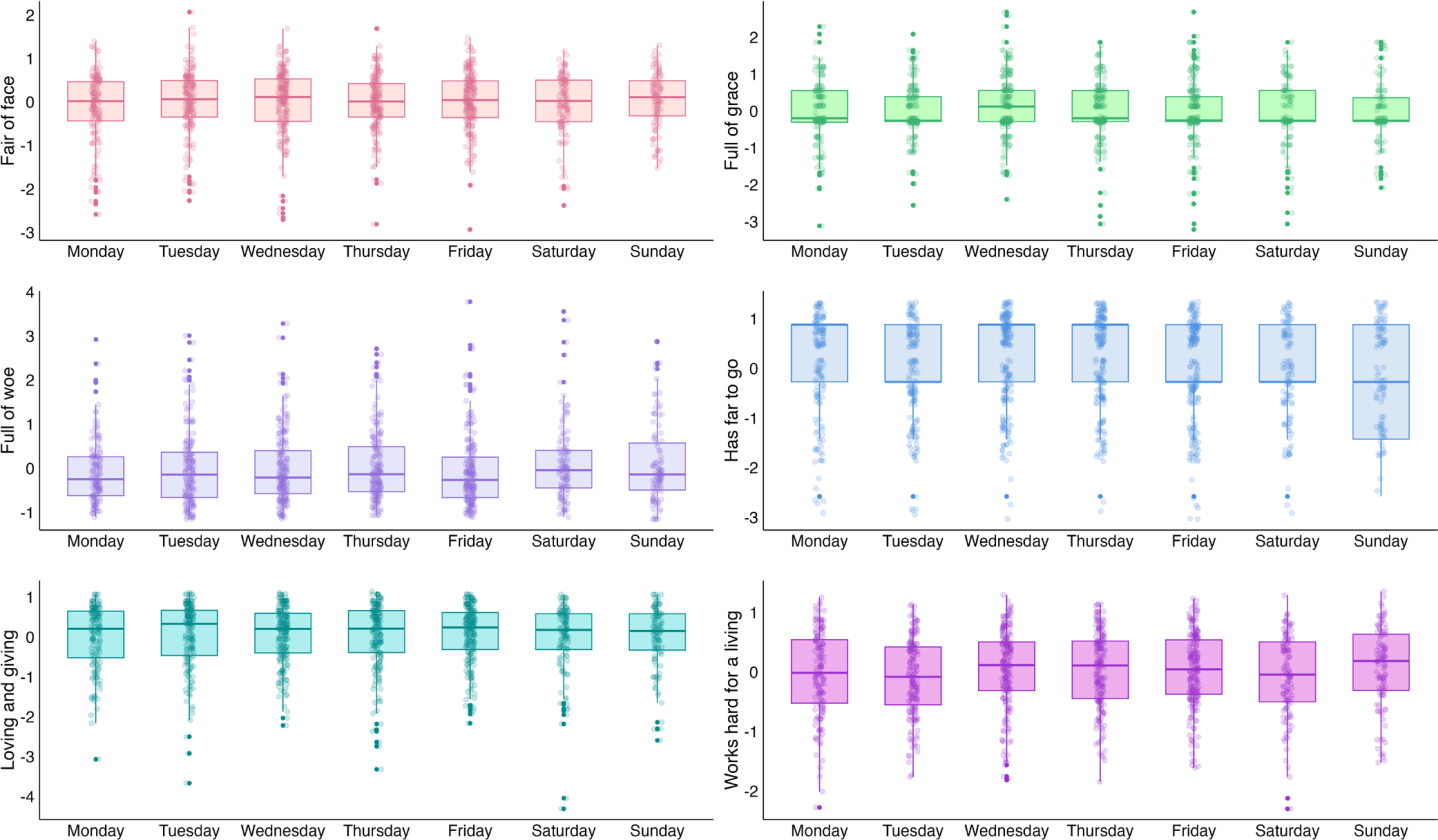
Boxplots of children's personality and physical traits by “Monday's Child” nursery rhyme, across days of the week of birth. Scores are plotted for one twin randomly selected from each pair. All plots show *z*‐scores (*M* = 0, SD = 1).

Day of the week of birth was not associated with any of the measures of children's personality and physical traits as forecast by the “Monday's Child” nursery rhyme (Figure 1). None of the six models reached statistical significance (*β* = −0.04 to 0.03, 95% CIs = [−0.10, 0.02] to [−0.02, 0.09], *p* = 0.195 to 0.754, *R* ^2^ = 0.00 for all models; full model results are shown in Table S3).

Adding the covariates of children's sex, birthweight, and SES improved model fit in all cases (*R* ^2^ ranged from 0.02 to 0.19; details in Table S4). However, day of birth continued to not be associated with children's differences in personality and physical traits.

### 3.2 Do Sunday's Children Experience More Favorable Outcomes Across Domains Than Children Born on Other Days?

Being born on a Sunday, compared to being born on another day of the week, did not predict children's personality and physical traits (*β* = −0.02 to 0.03, 95% CI = [−0.08, 0.03] to [−0.02, 0.09], *p* = 0.223 to 0.815, *R* ^2^ of 0.00 for all models; details in Table S5). Adding the covariates of children's sex, birthweight, and SES improved models' fit, but being born on a Sunday remained a nonsignificant predictor (*R* ^2^ ranged from 0.02 to 0.20, details in Table S6).

In addition, being born on a Sunday as compared to being born on another day of the week did not predict composite scores across all measures (*β* = 0.00, 95% CI = [−0.05, 0.05], *p* = 0.922, *R* ^2^ = 0.00; details in Table S7), rejecting the hypothesis of cumulative effects across domains. Including the covariates of children's sex, birthweight, and SES improved the model's fit, but being born on a Sunday remained a nonsignificant predictor (*R* ^2^ = 0.17, details in Table S7).

**FIGURE 2:**
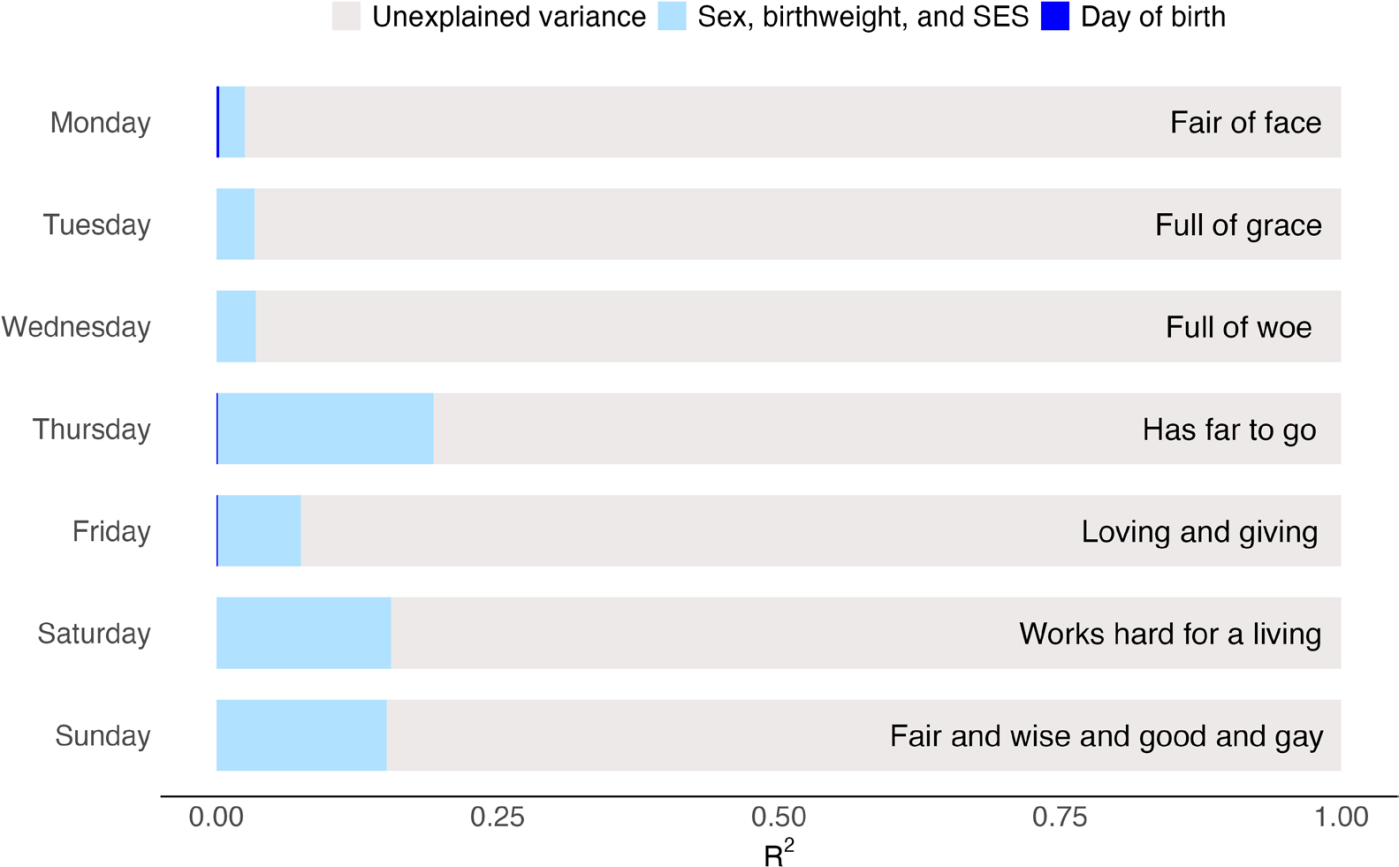
Predicting children's personality and physical traits from day of birth according to the “Monday's Child” rhyme. The *R* ^2^ for “Fair and wise and good and gay” was taken from the model for composite scores across all measures of children's personality and physical traits. SES, socioeconomic status.

The difference in prediction effect sizes between day of birth and the covariates of children's sex, birthweight, and SES across children's personality and physical traits are shown in Figure 2.

We conducted additional (not preregistered) analyses to fit our models separately to boys and girls, to establish whether predictions of traits by the day of birth varied as a function of sex. We found that the day of birth predicted personality traits neither in boys nor in girls. The results of these additional analyses can be found in the Supporting Information (see Tables S9–S13).

## 4 Discussion

We found no evidence for the predictive validity of children's day of the week of birth for their personality and physical traits between the ages of 5 and 18 years. The results were consistent across all weekdays and Sunday, which, despite being painted as the most auspicious day, was not associated with more favorable child outcomes in any domain. Overall, our findings suggest that the messages conveyed by the “Monday's Child” nursery rhyme do not manifest in observable differences in children's traits and behaviors.

Our study offers three distinct contributions to the literature. First, we add to the body of empirical studies that tested the benefits of engaging in nursery rhymes for children's language, literacy, and cognition (e.g., Bryant et al. 1989; Dunst et al. 2011; Starr, Oginni, and von Stumm 2024). Specifically, we addressed here whether the messages conveyed in nursery rhymes influenced children's personality development. Our findings suggest that this is not the case, at least for the “Monday's Child” nursery rhyme. We therefore conclude that the messages of nursery rhymes are unlikely to have perversive, long‐term influences on children's personality development. We hasten to add that this conclusion does not rule out that other aspects of nursery rhymes (e.g., alliteration, imaginative imagery) may affect children's development (e.g., language; Bryant et al. 1989).

Second, our research adds to the literature on the manifestation of prophecies about the day or time of birth. The results from our analyses of a British family cohort study diverge from earlier findings that were reported in Chinese and Ghanaian samples. In these populations, prophecies about the day or time of birth were found to be statistically significant predictors of children's developmental differences (Jahoda 1954; Mocan and Yu 2020). The discrepancy between the current and previous findings may be due to differences in the cultural valence countries assign to the day or time of birth. For example, in Chinese populations, the zodiac year of birth carries such significance that birth rates increase during the most auspicious zodiac year, the Dragon year, when compared to other years (Goodkind 1991; Nye and Xue 2014). Although the “Monday's Child” nursery rhyme is popular in British culture, it does not hold valence to the same degree as zodiac years do in Chinese culture.

Third, we found that our covariates of sex, birthweight, and socioeconomic status (SES) explained more variance in children's personality and physical traits than their day of the week of birth. A vast body of previous research has shown that sex, birthweight, and SES are robust predictors of children's social, cognitive, and educational outcomes (e.g., Bradley and Corwyn 2002; Buchmann et al. 2008; Jefferis et al. 2002), which our findings confirmed. In contrast, day of birth would likely only have had smaller, indirect effects on development, if any at all. We speculated that such effects, if they emerged, may be driven by confirmation bias, self‐fulfilling prophecies, or parents' influences on children's self‐image related to the “Monday's Child” nursery rhyme. However, our findings did not support the idea that the nursery rhyme altered parents' behavior or children's self‐image or behavior to the extent that children's personality would be influenced. Overall, we conclude that research on the covariate effects of sex, birthweight, and SES likely benefits our understanding of children's developmental differences to a greater extent than studies of children's day of the week of birth.

### 4.1 Strengths and Limitations

A key strength of this study lies in its longitudinal data from a UK population‐representative family cohort study of children for whom repeated multi‐measure, multi‐informant assessments of personality and physical traits were available across childhood and adolescence. However, the study also had some limitations.

First, no data were available on the extent to which the families were familiar with, engaged in, or adhered to the “Monday's Child” nursery rhyme. Thus, we could not ascertain whether our findings were confounded by family‐level differences in children's and parents' endorsement of the verses of the “Monday's Child” rhyme. Familiarity and engagement were key to our rationale of why the “Monday's Child” nursery rhyme may predict children's differences in personality and physical traits. We acknowledge that our research would have benefited from measures that directly assessed these constructs. That said, the omnipresence of the “Monday's Child” nursery rhyme in popular culture makes it likely that most families have been exposed to the rhyme in some capacity. Also, family‐level engagement with the nursery rhyme may have varied in our sample, but exposure to the rhyme could have also occurred through other pathways, including interactions with peers, teachers, and media.

Second, the study sample comprised twins, which may limit our study for two reasons. For one, twins differ from singleton births in prevalence (Smith et al. 2014), parenting demands (Olivennes et al. 2005), and early childhood developmental trajectories (Pulkkinen et al. 2003; Voracek and Haubner 2008). Consequently, twins' developmental experiences may differ from those of singletons, which could impair the generalisability of our present findings to singleton populations. For the other, twin births are more often than singletons induced or scheduled for a cesarean delivery before the twins' estimated due date (Hartley et al. 2001). Some might argue that, in these cases, twins are not being born on their intended birthday that could predict their personality and physical traits. This limitation would affect the validity of our findings if there was an effect of the day of week of birth on children's personality differences that was not transmitted via confirmation bias, self‐fulfulling prophecies, or parenting, as we speculated. Such effects are implied by astrology, for instance, where the position of the sun, moon, and planets at the time of birth are said to predict later personality traits (Thagard 1978). Yet, astrological predictions have been deemed pseudoscientific and are unsubstantiated (Clarke et al. 1996; Hartmann et al. 2006; Thagard 1978). Our study does not claim that the day of birth had predictive powers that were independent of established psychological mechanisms. In any case, days of birth often deviate from the estimated “due date” in both twin and singleton births for a variety of reasons (e.g., preterm, late term, or induction).

Third, it may be that the impact of “Monday's Child” on children varies as a function of changes in the nursery rhyme's popularity over time. Our null findings pertain to a cohort of children born in the mid‐90s (i.e., Millennials; Powell et al. 2017). However, associations between day of the birth and children's personality and physical traits may be evident in other generations. For example, the public's exposure to the “Monday's Child” nursery rhyme has likely increased since the release of the high‐profile 2022 Netflix series “Wednesday”. Its messages may therefore shape the socialization experiences and development of the current Generation Alpha (born between 2013 and 2025) to a greater extent than of previous generations (e.g., Millennials and Generation Z).

## 5 Conclusion

Wednesday's child is no more woeful than Monday's child is fair. Although children vary greatly in their personality and physical traits, their developmental differences are unlikely to be affected by the day of the week on which they were born. However, children's differences in personality are predicted by their sex, birthweight, and socioeconomic status.

## Author Contributions

E.W., A.B., K.W., F.A.R.O., and S.v.S. conceived the study. E.W. lead on the statistical analysis and writing. All authors reviewed and commented on the manuscript.

## Disclosure

Preregistration: The hypotheses, methods, and the analysis plan were preregistered (https://sites.duke.edu/moffittcaspiprojects/files/2024/05/Wood_2024_Day‐of‐birth‐and‐outcomes.pdf) on 8 May 2024, prior to requesting data access which was granted on 14 May 2024. There were minor deviations from the preregistration (for details see the Supporting Information).

## Ethics Statement

The Joint South London and Maudsley and the Institute of Psychiatry Research Ethics Committee approved each phase of the E‐Risk study (NRES 1997/122). Parents gave informed consent and twins gave assent before age 18 and consent at age 18.

## Conflicts of Interest

The authors declare no conflicts of interest.

## Supporting information


Data S1

## Data Availability

The E‐Risk Study operate a managed access process to protect the privacy of their participants and the integrity of their study. All primary data and study materials are available upon request to the E‐Risk study steering committee (https://eriskstudy.com/data‐access/). All scripts can be found here: osf.io./qxv4z.
