# Supplementary material for: Are Wednesday's Children Full of Woe? Children's Differences in Personality Are Independent of Day of Birth
Source: J Pers. 2025 Jul 11;94(3):370–9. doi: 10.1111/jopy.70005 (PMC12340769; doi:10.1111/jopy.70005)
Supplement: Supplementary file 1 — Data S1 [file JOPY-94-370-s001.docx]

**Supplementary Materials**

**Are Wednesday’s children full of woe? Childhood differences in personality and physical traits are independent of day of birth**

Table of Contents

[Table S1: Descriptive statistics of study variables 2](#_Toc200798074)

[Table S2: Sample descriptors 3](#_Toc200798075)

[Figure S1: Family socioeconomic status at age 5 by day of birth. 4](#_Toc200798076)

[Figure S2: Birthweight of sample by day of birth 5](#_Toc200798077)

[Figure S3: Sex of sample by day of birth 6](#_Toc200798078)

[Table S3: Day of birth as a predictor of children’s personality and physical traits. 7](#_Toc200798079)

[Table S4: Day of birth, sex, SES, and birthweight as predictors of children’s personality and physical traits. 8](#_Toc200798080)

[Table S5: Sunday birth as a predictor of children’s personality and physical traits 9](#_Toc200798081)

[Table S6: Sunday birth, sex, SES, and birthweight as predictors of personality and physical traits 10](#_Toc200798082)

[Table S7: Sunday birth, and Sunday birth, sex, SES, and birthweight, as predictors of all personality and physical traits 11](#_Toc200798083)

[Table S8: Single ‘well-poised, gracious, and confident’ rating as an alternative measure of grace 12](#_Toc200798084)

[Table S9: Day of birth, SES, and birthweight as predictors of children’s personality and physical traits in female participants. 13](#_Toc200798085)

[Table S10: Day of birth, SES, and birthweight as predictors of children’s personality and physical traits in male participants. 14](#_Toc200798086)

[Table S11: Sunday birth, SES, and birthweight as predictors of personality and physical traits in females 15](#_Toc200798087)

[Table S12: Sunday birth, SES, and birthweight as predictors of personality and physical traits in males 16](#_Toc200798088)

[Table S13: Sunday birth, SES, and birthweight as predictors of all personality and physical traits in female participants 17](#_Toc200798089)

[Table S14: Sunday birth, SES, and birthweight as predictors of all personality and physical traits in male participants 18](#_Toc200798090)

[Deviations from the pre-registration 19](#_Toc200798091)

##

## **Table S1:** **Descriptive statistics of study variables**

|  | N | Missing | Mean | SD | Min | Max | Skew | Kurt | n | α |
| --- | --- | --- | --- | --- | --- | --- | --- | --- | --- | --- |
| Birthweight (g) | 1039 | 77 | 2438.23 | 536.59 | 454.00 | 4114.38 | -0.45 | 3.43 | - | - |
| Prosocial behaviour | 1116 | 0 | 0.02 | 0.80 | -4.31 | 1.14 | -1.25 | 5.07 | 4 | .80 |
| PE score | 1094 | 22 | 0.00 | 0.82 | -3.22 | 2.68 | -0.21 | 4.32 | 3 | .64 |
| Emotionality | 1116 | 0 | 0.00 | 0.81 | -1.15 | 3.78 | 1.28 | 5.00 | 4 | .84 |
| Hardworking | 1113 | 3 | 0.00 | 0.66 | -2.30 | 1.35 | -0.49 | 2.80 | 9 | .82 |
| Attractiveness | 1116 | 0 | -0.02 | 0.71 | -2.93 | 2.07 | -0.84 | 4.26 | 4 | .61 |
| Educational achievement | 1031 | 85 | 0.00 | 0.99 | -2.58 | 0.88 | -0.74 | 2.41 | 1 | - |
| Outcome composite | 1116 | 0 | -0.01 | 0.48 | -2.31 | 1.04 | -0.85 | 4.02 | 6 | - |
| Graciousness | 1033 | 83 | -0.03 | 1.01 | -1.83 | 0.90 | -0.59 | 2.03 | 1 | - |

Note: Descriptive were based on one randomly selected twin per pair. n refers to the number of items included in each variable. α refers to Cronbach’s Alpha. PE = Physical education. Outcome Composite = Composite score of all personality and physical traits tested, excluding graciousness. Graciousness describes an alternative measure of grace, analysed and reported separately.

## **Table S2: Sample descriptors**

|  | **Overall (N = 1116 twin pairs)** | |
| --- | --- | --- |
|  | N (%) | |
| Day of Birth | |  |
| Monday | 146 (13.1) | |
| Tuesday | 168 (15.1) | |
| Wednesday | 194 (17.4) | |
| Thursday | 182 (16.3) | |
| Friday | 202 (18.1) | |
| Saturday | 119 (10.7) | |
| Sunday | 105 (9.4) | |
| Sex |  | |
| Male | 546 (48.9) | |
| Female | 570 (51.1) | |
| Zygosity |  | |
| MZ | 621 (55.6) | |
| DZ | 495 (44.4) | |
| SES |  | |
| Low | 371 (33.2) | |
| Mid | 369 (33.1) | |
| High | 376 (33.7) | |

Note: The table refers to twin pairs. MZ = Monozygotic. DZ = Dizygotic. SES = socioeconomic status at age 5, including educational attainment, occupation, and household income.


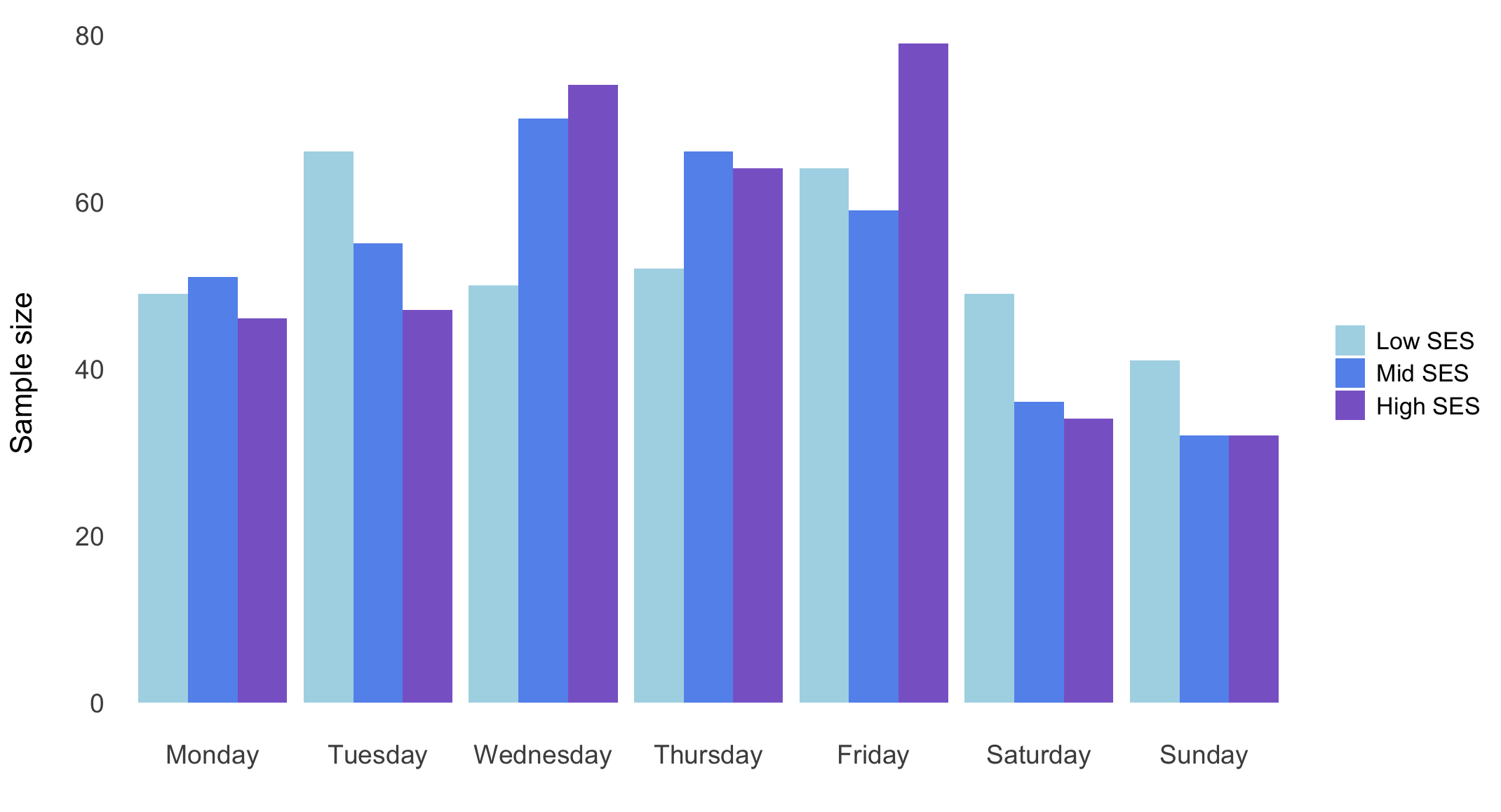


## **Figure S1: Family socioeconomic status at age 5 by day of birth.**

Note: Estimates are based on twin pairs. SES = socioeconomic status, including educational attainment, occupation, and household income.


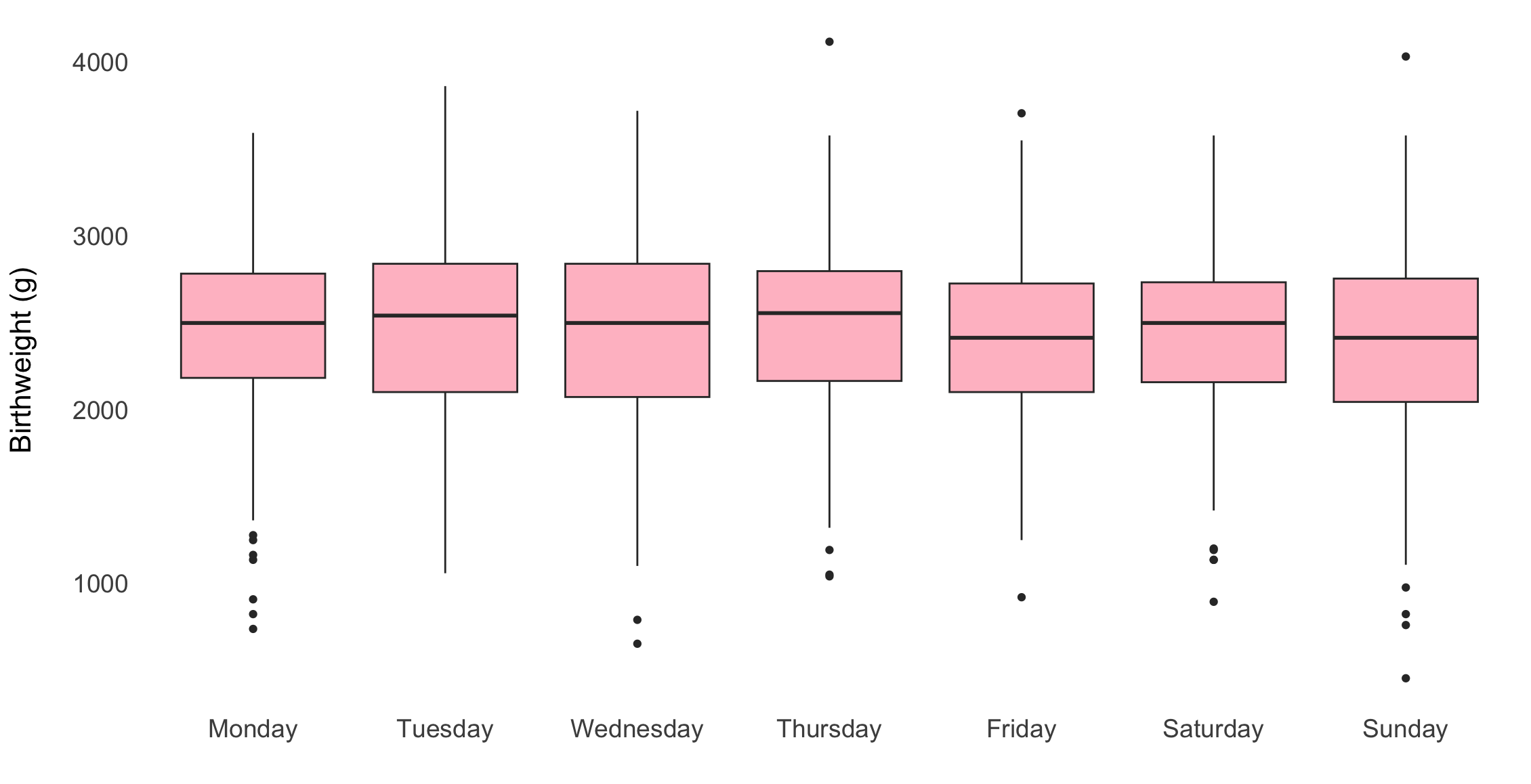


## **Figure S2: Birthweight of sample by day of birth**

Note: Estimates are based on one randomly selected twin from each twin pair.


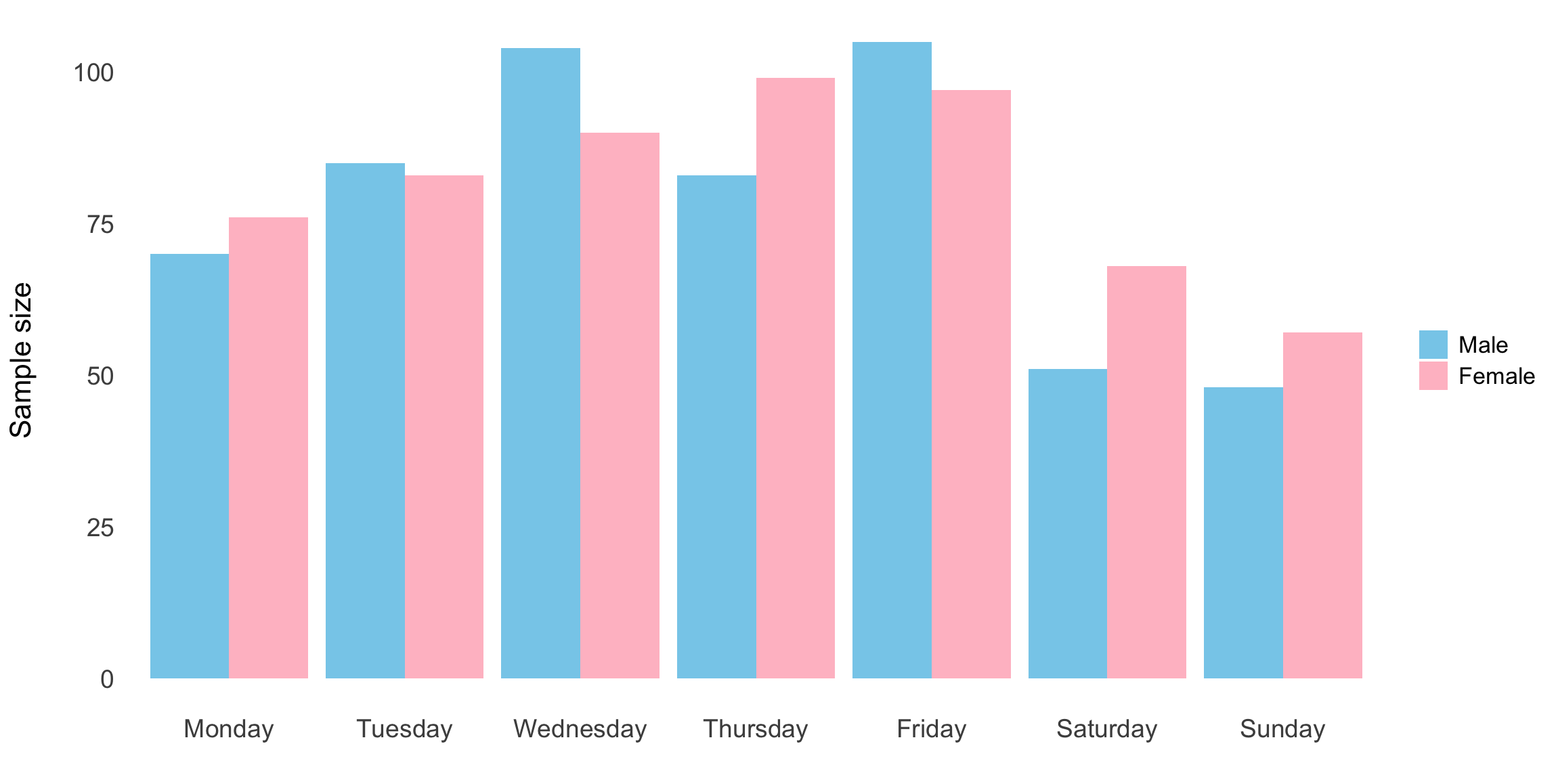


## **Figure S3: Sex of sample by day of birth**

Note: Estimates are based on twin pairs.

## **Table S3: Day of birth as a predictor of children’s personality and physical traits.**

| Model | Est | β | SE | 95% CI | *p* | *R^2^* |
| --- | --- | --- | --- | --- | --- | --- |
| Attractiveness ~ Monday | -.08 | -.04 | 0.03 | [-0.10, 0.02] | .202 | .00 |
| PE score ~ Tuesday | -.02 | -.01 | 0.03 | [-0.06, 0.05] | .754 | .00 |
| Emotional problems ~ Wednesday | -.05 | -.02 | 0.03 | [-0.07, 0.03] | .388 | .00 |
| Educational attainment ~ Thursday | .08 | .03 | 0.03 | [-0.02, 0.09] | .265 | .00 |
| Prosocial behaviour ~ Friday | .07 | .03 | 0.03 | [-0.02, 0.08] | .195 | .00 |
| Hardworking ~ Saturday | -.02 | -.01 | 0.03 | [-0.07, 0.05] | .698 | .00 |

Note: Est = unadjusted estimate. β = standardised regression coefficient. SE = standard error. 95% CI = 95% confidence interval. PE = physical education. Standard errors were clustered at the family level to account for the non-independence of observations (i.e., two twins per family).

## **Table S4: Day of birth, sex, SES, and birthweight as predictors of children’s personality and physical traits.**

| Model | Est | β | SE | 95% CI | *p* | *R²* |
| --- | --- | --- | --- | --- | --- | --- |
| Attractiveness ~ Monday | -.07 | -.04 | 0.03 | [-0.09, 0.02] | .228 | .02 |
| + sex | .13 | .09 | 0.03 | [0.04, 0.15] | .001** |  |
| + SES | .06 | .08 | 0.03 | [0.03, 0.14] | .002** |  |
| + birthweight | .05 | .08 | 0.03 | [0.01, 0.14] | .015* |  |
| PE score ~ Tuesday | -.00 | .00 | 0.03 | [-0.06, 0.05] | .955 | .03 |
| + sex | .03 | .02 | 0.03 | [-0.03, 0.07] | .461 |  |
| + SES | .12 | .14 | 0.03 | [0.09, 0.19] | <.001*** |  |
| + birthweight | .09 | .11 | 0.03 | [0.05, 0.17] | <.001*** |  |
| Emotional problems ~ Wednesday | -.02 | -.01 | 0.03 | [-0.06, 0.04] | .695 | .04 |
| + sex | -.01 | -.01 | 0.03 | [-0.06, 0.05] | .825 |  |
| + SES | -.15 | -.19 | 0.03 | [-0.24, -0.14] | <.001*** |  |
| + birthweight | .00 | .00 | 0.02 | [-0.04, 0.05] | .861 |  |
| Educational achievement ~ Thursday | .03 | .01 | 0.02 | [-0.04, 0.06] | .643 | .19 |
| + sex | .17 | .08 | 0.03 | [0.03, 0.14] | .001** |  |
| + SES | .43 | .43 | 0.02 | [0.38, 0.47] | <.001*** |  |
| + birthweight | .02 | .02 | 0.03 | [-0.03, 0.08] | .386 |  |
| Prosocial behaviour ~ Friday | .08 | .04 | 0.03 | [-0.01, 0.09] | .133 | .07 |
| + sex | .40 | .25 | 0.03 | [0.20, 0.30] | <.001*** |  |
| + SES | .08 | .10 | 0.03 | [0.05, 0.16] | <.001*** |  |
| + birthweight | .03 | .04 | 0.03 | [-0.01, 0.09] | .134 |  |
| Hardworking ~ Saturday | -.02 | -.01 | 0.03 | [-0.06, 0.04] | .708 | .16 |
| + sex | .41 | .31 | 0.02 | [0.26, 0.36] | <.001*** |  |
| + SES | .16 | .24 | 0.03 | [0.19, 0.29] | <.001*** |  |
| + birthweight | .02 | .03 | 0.03 | [-0.02, 0.08] | .197 |  |

Note: Est = unadjusted estimate. β = standardised regression coefficient. SE = standard error. 95% CI = 95% confidence interval. SES = socioeconomic status, including educational attainment, occupation, and household income. Standard errors were clustered at the family level to account for the non-independence of observations (i.e., two twins per family). **p*<.05. ***p*<.01. ****p*<.001.

## **Table S5: Sunday birth as a predictor of children’s personality and physical traits**

| Model | Est | β | SE | 95% CI | *p* | *R²* |
| --- | --- | --- | --- | --- | --- | --- |
| Attractiveness ~ Sunday | .05 | .02 | 0.02 | [-0.03, 0.07] | .384 | .00 |
| PE score ~ Sunday | -.05 | -.02 | 0.03 | [-0.07, 0.03] | .441 | .00 |
| Emotional problems ~ Sunday | .06 | .02 | 0.03 | [-0.03, 0.07] | .413 | .00 |
| Educational achievement ~ Sunday | -.07 | -.02 | 0.03 | [-0.08, 0.03] | .446 | .00 |
| Prosocial behaviour ~ Sunday | .02 | .01 | 0.02 | [-0.04, 0.05] | .815 | .00 |
| Hardworking ~ Sunday | .08 | .03 | 0.03 | [-0.02, 0.09] | .223 | .00 |

Note: Est = unadjusted estimate. β = standardised regression coefficient. SE = standard error. 95% CI = 95% confidence interval. Standard errors were clustered at the family level to account for the non-independence of observations (i.e., two twins per family). To account for multiple comparisons, the alpha level was adjusted to (6/0.05) 0.01, so that *p*<.01 was the new p-value threshold for significance.

## **Table S6: Sunday birth, sex, SES, and birthweight as predictors of personality and physical traits**

| Model | Est | β | SE | 95% CI | *p* | *R²* |
| --- | --- | --- | --- | --- | --- | --- |
| Attractiveness ~ Sunday | .06 | .03 | 0.02 | [-0.02, 0.07] | .282 | .02 |
| + sex | .13 | .09 | 0.03 | [0.04, 0.14] | .001** |  |
| + SES | .06 | .08 | 0.03 | [0.03, 0.14] | .001** |  |
| + birthweight | .06 | .08 | 0.03 | [0.02, 0.14] | .006** |  |
| PE score ~ Sunday | -.02 | -.01 | 0.02 | [-0.05, 0.04] | .742 | .04 |
| + sex | .03 | .02 | 0.03 | [-0.03, 0.07] | .490 |  |
| + SES | .12 | .14 | 0.03 | [0.09, 0.19] | <.001*** |  |
| + birthweight | .09 | .11 | 0.03 | [0.06, 0.16] | <.001*** |  |
| Emotional problems ~ Sunday | .04 | .01 | 0.02 | [-0.03, 0.06] | .572 | .04 |
| + sex | .00 | .00 | 0.02 | [-0.05, 0.05] | .981 |  |
| + SES | -.16 | -.19 | 0.02 | [-0.24, -0.14] | <.001*** |  |
| + birthweight | -.01 | -.01 | 0.02 | [-0.05, 0.04] | .804 |  |
| Educational achievement ~ Sunday | -.03 | -.01 | 0.02 | [-0.05, 0.04] | .750 | .20 |
| + sex | .19 | .10 | 0.02 | [0.05, 0.14] | <.001*** |  |
| + SES | .43 | .43 | 0.02 | [0.39, 0.48] | <.001*** |  |
| + birthweight | .02 | .02 | 0.03 | [-0.03, 0.08] | .351 |  |
| Prosocial behaviour ~ Sunday | .02 | .01 | 0.02 | [-0.04, 0.05] | .811 | .07 |
| + sex | .39 | .24 | 0.02 | [0.19, 0.29] | <.001*** |  |
| + SES | .08 | .10 | 0.03 | [0.05, 0.15] | <.001*** |  |
| + birthweight | .03 | .03 | 0.02 | [-0.02, 0.08] | .177 |  |
| Hardworking ~ Sunday | .09 | .04 | 0.02 | [-0.01, 0.09] | .126 | .16 |
| + sex | .42 | .32 | 0.02 | [0.27, 0.36] | <.001*** |  |
| + SES | .16 | .24 | 0.02 | [0.19, 0.29] | <.001*** |  |
| + birthweight | .02 | .03 | 0.02 | [-0.01, 0.08] | .168 |  |

Note: Est = unadjusted estimate. β = standardised regression coefficient. SE = standard error. 95% CI = 95% confidence interval. SES = socioeconomic status, including educational attainment, occupation, and household income. Standard errors were clustered at the family level to account for the non-independence of observations (i.e., two twins per family). To account for multiple comparisons, the alpha level was adjusted to (6/0.05) 0.01, so that *p*<.01 was the new p-value threshold for significance. **p<.01, ****p*<.001.

## **Table S7: Sunday birth, and Sunday birth, sex, SES, and birthweight, as predictors of all personality and physical traits**

| Model | Est | β | SE | 95% CI | *p* | *R²* |
| --- | --- | --- | --- | --- | --- | --- |
| Outcome composite ~ Sunday | .00 | .00 | 0.02 | [-0.05, 0.05] | .922 | .00 |
| Outcome composite ~ Sunday | .02 | .01 | 0.02 | [-0.03, 0.05] | .657 |  |
| + sex | .19 | .21 | 0.02 | [0.16, 0.25] | <.001*** | .17 |
| + SES | .16 | .35 | 0.02 | [0.31, 0.39] | <.001*** |  |
| + birthweight | .04 | .08 | 0.03 | [0.03, 0.14] | .002** |  |

Note: Est = unadjusted estimate. β = standardised regression coefficient. SE = standard error. 95% CI = 95% confidence interval. SES = socioeconomic status, including educational attainment, occupation, and household income. Standard errors were clustered at the family level to account for the non-independence of observations (i.e., two twins per family). ***p*<.01 ****p*<.001.

## **Table S8: Single ‘well-poised, gracious, and confident’ rating as an alternative measure of grace**

| Model | Est | β | SE | 95% CI | *p* | *R²* |
| --- | --- | --- | --- | --- | --- | --- |
| Graciousness ~ Tuesday | -.06 | -.02 | 0.03 | [-0.08, 0.03] | .392 | .00 |
| Graciousness ~ Tuesday | -.01 | -.00 | 0.03 | [-0.05, 0.05] | .928 | .06 |
| + sex | .10 | .05 | 0.02 | [0.00, 0.10] | .041* |  |
| + SES | .24 | .24 | 0.02 | [0.19, 0.29] | <.001*** |  |
| + birthweight | .03 | .02 | 0.02 | [-0.02, 0.07] | .299 |  |
| Graciousness ~ Sunday | .00 | .00 | 0.03 | [-0.05, 0.05] | .984 | .00 |
| Graciousness ~ Sunday | .03 | .01 | 0.02 | [-0.04, 0.06] | .706 | .06 |
| + sex | .11 | .06 | 0.02 | [0.01, 0.10] | .015* |  |
| + SES | .24 | .24 | 0.02 | [0.19, 0.29] | <.001*** |  |
| + birthweight | .04 | .04 | 0.02 | [0.01, 0.08] | .107 |  |

Note: Est = unadjusted estimate. β = standardised regression coefficient. SE = standard error. 95% CI = 95% confidence interval. SES = socioeconomic status, including educational attainment, occupation, and household income. Standard errors were clustered at the family level to account for the non-independence of observations (i.e., two twins per family). **p*<.05 ****p*< .001.

## **Table S9: Day of birth, SES, and birthweight as predictors of children’s personality and physical traits in female participants.**

| Model | Est | β | SE | 95% CI | *p* | *R²* |
| --- | --- | --- | --- | --- | --- | --- |
| Attractiveness ~ Monday | -.04 | -.02 | 0.04 | [-0.10, 0.06] | .638 | .00 |
| + SES | .03 | .04 | 0.04 | [-0.03, 0.12] | .265 |  |
| + birthweight | .02 | .02 | 0.04 | [-0.07, 0.11] | .624 |  |
| PE Score ~ Tuesday | .00 | .00 | 0.04 | [-0.08, 0.08] | .997 | .03 |
| + SES | .12 | .14 | 0.04 | [0.06, 0.22] | <.001*** |  |
| + birthweight | .07 | .08 | 0.04 | [0.00, 0.16] | .043* |  |
| Graciousness ~ Tuesday | -.01 | .00 | 0.04 | [-0.08, 0.07] | .906 | .03 |
| + SES | .17 | .18 | 0.04 | [0.11, 0.25] | <.001*** |  |
| + birthweight | .04 | .04 | 0.03 | [-0.03, 0.10] | .248 |  |
| Emotional problems ~ Wednesday | -.08 | -.04 | 0.03 | [-0.11, 0.03] | .265 | .03 |
| + SES | -.12 | -.15 | 0.04 | [-0.22, -0.08] | <.001*** |  |
| + birthweight | -.01 | -.01 | 0.03 | [-0.08, 0.06] | .742 |  |
| Educational achievement ~ Thursday | .02 | .01 | 0.03 | [-0.06, 0.07] | .841 | .17 |
| + SES | .39 | .41 | 0.04 | [0.34, 0.48] | <.001*** |  |
| + birthweight | .02 | .02 | 0.04 | [-0.06, 0.09] | .677 |  |
| Prosocial behaviour ~ Friday | .03 | .02 | 0.04 | [-0.05, 0.09] | .605 | .01 |
| + SES | .07 | .10 | 0.04 | [0.02, 0.18] | .011* |  |
| + birthweight | .03 | .04 | 0.04 | [-0.03, 0.11] | .292 |  |
| Hardworking ~ Saturday | -.05 | -.03 | 0.04 | [-0.11, 0.06] | .515 | .07 |
| + SES | .15 | .26 | 0.04 | [0.19, 0.33] | <.001*** |  |
| + birthweight | .00 | .01 | 0.04 | [-0.07, 0.08] | .874 |  |

Note: Est = unadjusted estimate. β = standardised regression coefficient. SE = standard error. 95% CI = 95% confidence interval. SES = socioeconomic status, including educational attainment, occupation, and household income. Standard errors were clustered at the family level to account for the non-independence of observations (i.e., two twins per family). **p*<.05. ***p*<.01. ****p*<.001.

## **Table S10: Day of birth, SES, and birthweight as predictors of children’s personality and physical traits in male participants.**

| Model | Est | β | SE | 95% CI | *p* | *R²* |
| --- | --- | --- | --- | --- | --- | --- |
| Attractiveness ~ Monday | -.10 | -.05 | 0.04 | [-0.13, 0.03] | .231 | .04 |
| + SES | .09 | .13 | 0.04 | [0.05, 0.20] | .001** |  |
| + birthweight | .09 | .13 | 0.04 | [0.04, 0.21] | .003** |  |
| PE Score ~ Tuesday | -.02 | -.01 | 0.04 | [-0.08, 0.06] | .819 | .04 |
| + SES | .12 | .14 | 0.04 | [0.07, 0.21] | <.001*** |  |
| + birthweight | .12 | .13 | 0.04 | [0.06, 0.21] | .001** |  |
| Graciousness ~ Tuesday | -.02 | -.01 | 0.04 | [-0.08, 0.07] | .831 | .10 |
| + SES | .32 | .31 | 0.03 | [0.24, 0.38] | <.001*** |  |
| + birthweight | .01 | .01 | 0.04 | [-0.06, 0.08] | .790 |  |
| Emotional problems ~ Wednesday | .03 | .01 | 0.04 | [-0.06, 0.09] | .685 | .05 |
| + SES | -.19 | -.22 | 0.04 | [-0.29, -0.15] | <.001*** |  |
| + birthweight | .02 | .02 | 0.04 | [-0.05, 0.09] | .576 |  |
| Educational achievement ~ Thursday | .06 | .02 | 0.03 | [-0.05, 0.09] | .531 | .21 |
| + SES | .47 | .45 | 0.03 | [0.39, 0.52] | <.001*** |  |
| + birthweight | .03 | .03 | 0.04 | [-0.05, 0.11] | .437 |  |
| Prosocial behaviour ~ Friday | .11 | .06 | 0.04 | [-0.02, 0.13] | .129 | .02 |
| + SES | .10 | .11 | 0.04 | [0.04, 0.19] | .003** |  |
| + birthweight | .03 | .04 | 0.04 | [-0.03, 0.12] | .289 |  |
| Hardworking ~ Saturday | .02 | .01 | 0.04 | [-0.07, 0.08] | .856 | .07 |
| + SES | .18 | .25 | 0.04 | [0.18, 0.33] | <.001*** |  |
| + birthweight | .04 | .06 | 0.04 | [-0.02, 0.13] | .119 |  |

Note: Est = unadjusted estimate. β = standardised regression coefficient. SE = standard error. 95% CI = 95% confidence interval. SES = socioeconomic status, including educational attainment, occupation, and household income. Standard errors were clustered at the family level to account for the non-independence of observations (i.e., two twins per family). ***p*<.01. ****p*<.001.

## **Table S11: Sunday birth, SES, and birthweight as predictors of personality and physical traits in females**

| Model | Est | β | SE | 95% CI | *p* | *R²* |
| --- | --- | --- | --- | --- | --- | --- |
| Attractiveness ~ Sunday | .03 | .01 | 0.04 | [-0.06, 0.08] | .754 |  |
| + SES | .02 | .03 | 0.04 | [-0.04, 0.11] | .374 | .00 |
| + birthweight | .02 | .03 | 0.04 | [-0.05, 0.11] | .480 |  |
| PE Score ~ Sunday | -.05 | -.02 | 0.04 | [-0.09, 0.05] | .609 |  |
| + SES | .11 | .15 | 0.04 | [0.07, 0.22] | <.001*** | .03 |
| + birthweight | .06 | .08 | 0.04 | [0.01, 0.16] | .033* |  |
| Graciousness ~ Sunday | .09 | .03 | 0.03 | [-0.03, 0.09] | .395 |  |
| + SES | .18 | .18 | 0.03 | [0.12, 0.25] | <.001*** | .04 |
| + birthweight | .04 | .04 | 0.03 | [-0.02, 0.10] | .192 |  |
| Emotional problems ~ Sunday | .08 | .03 | 0.04 | [-0.04, 0.10] | .391 |  |
| + SES | -.13 | -.16 | 0.03 | [-0.23, -0.10] | <.001*** | .03 |
| + birthweight | -.02 | -.02 | 0.03 | [-0.09, 0.04] | .488 |  |
| Educational achievement ~ Sunday | .09 | .03 | 0.03 | [-0.04, 0.09] | .435 |  |
| + SES | .39 | .41 | 0.03 | [0.35, 0.48] | <.001*** | .17 |
| + birthweight | .02 | .02 | 0.04 | [-0.05, 0.10] | .521 |  |
| Prosocial behaviour ~ Sunday | -.02 | -.01 | 0.03 | [-0.07, 0.06] | .839 |  |
| + SES | .06 | .09 | 0.04 | [0.02, 0.16] | .016* | .01 |
| + birthweight | .03 | .04 | 0.04 | [-0.03, 0.11] | .247 |  |
| Hardworking ~ Sunday | .14 | .07 | 0.03 | [0.01, 0.14] | .034* |  |
| + SES | .15 | .25 | 0.03 | [0.19, 0.32] | <.001*** | .07 |
| + birthweight | .00 | .00 | 0.04 | [-0.07, 0.08] | .920 |  |

Note: Est = unadjusted estimate. β = standardised regression coefficient. SE = standard error. 95% CI = 95% confidence interval. SES = socioeconomic status, including educational attainment, occupation, and household income. Standard errors were clustered at the family level to account for the non-independence of observations (i.e., two twins per family). To account for multiple comparisons, the alpha level was adjusted to (6/0.05) 0.01, so that *p*<.01 was the new p-value threshold for significance. *p<.05. ****p*<.001.

## **Table S12: Sunday birth, SES, and birthweight as predictors of personality and physical traits in males**

| Model | Est | β | SE | 95% CI | *p* | *R²* |
| --- | --- | --- | --- | --- | --- | --- |
| Attractiveness ~ Sunday | .09 | .04 | 0.03 | [-0.02, 0.10] | .221 |  |
| + SES | .09 | .14 | 0.04 | [0.07, 0.21] | <.001*** | .04 |
| + birthweight | .09 | .13 | 0.04 | [0.05, 0.21] | .001** |  |
| PE Score ~ Sunday | -.00 | .00 | 0.03 | [-0.06, 0.06] | .999 |  |
| + SES | .12 | .14 | 0.04 | [0.07, 0.21] | <.001*** | .04 |
| + birthweight | .11 | .13 | 0.04 | [0.06, 0.21] | .001** |  |
| Graciousness ~ Sunday | -.03 | -.01 | 0.04 | [-0.08, 0.06] | .814 |  |
| + SES | .31 | .30 | 0.03 | [0.24, 0.37] | <.001*** | .09 |
| + birthweight | .03 | .03 | 0.03 | [-0.04, 0.10] | .351 |  |
| Emotional problems ~ Sunday | -.02 | -.01 | 0.03 | [-0.07, 0.06] | .844 |  |
| + SES | -.19 | -.02 | 0.03 | [-0.29, -0.15] | <.001*** | .05 |
| + birthweight | .01 | .01 | 0.03 | [-0.05, 0.08] | .684 |  |
| Educational achievement ~ Sunday | -.16 | -.04 | 0.03 | [-0.11, 0.02] | .172 |  |
| + SES | .48 | .46 | 0.03 | [0.40, 0.52] | <.001*** | .22 |
| + birthweight | .03 | .03 | 0.04 | [-0.05, 0.10] | .475 |  |
| Prosocial behaviour ~ Sunday | .06 | .02 | 0.04 | [-0.06, 0.09] | .622 |  |
| + SES | .09 | .11 | 0.04 | [0.04, 0.18] | .003** | .01 |
| + birthweight | .02 | .03 | 0.04 | [-0.04, 0.10] | .443 |  |
| Hardworking ~ Sunday | .02 | .01 | 0.04 | [-0.07, 0.08] | .871 |  |
| + SES | .17 | .25 | 0.04 | [0.18, 0.32] | <.001*** | .07 |
| + birthweight | .04 | .06 | 0.04 | [-0.01, 0.13] | .075 |  |

Note: Est = unadjusted estimate. β = standardised regression coefficient. SE = standard error. 95% CI = 95% confidence interval. SES = socioeconomic status, including educational attainment, occupation, and household income. Standard errors were clustered at the family level to account for the non-independence of observations (i.e., two twins per family). To account for multiple comparisons, the alpha level was adjusted to (6/0.05) 0.01, so that *p*<.01 was the new p-value threshold for significance. ***p*<.01. ****p*<.001.

## **Table S13: Sunday birth, SES, and birthweight as predictors of all personality and physical traits in female participants**

| Model | Est | β | SE | 95% CI | *p* | *R²* |
| --- | --- | --- | --- | --- | --- | --- |
| Outcome composite ~ Sunday | .01 | .01 | 0.03 | [-0.05, 0.07] | .759 | .12 |
| + SES | .14 | .33 | 0.03 | [0.27, 0.40] | <.001*** |  |
| + birthweight | .03 | .06 | 0.04 | [-0.01, 0.14] | .094 |  |

Note: Est = unadjusted estimate. β = standardised regression coefficient. SE = standard error. 95% CI = 95% confidence interval. SES = socioeconomic status, including educational attainment, occupation, and household income. Standard errors were clustered at the family level to account for the non-independence of observations (i.e., two twins per family). ****p*<.001.

## **Table S14: Sunday birth, SES, and birthweight as predictors of all personality and physical traits in male participants**

| Model | Est | β | SE | 95% CI | *p* | *R²* |
| --- | --- | --- | --- | --- | --- | --- |
| Outcome composite ~ Sunday | .02 | .01 | 0.03 | [-0.05, 0.07] | .776 | .16 |
| + SES | .19 | .38 | 0.03 | [0.32, 0.44] | <.001*** |  |
| + birthweight | .05 | .10 | 0.04 | [0.02, 0.18] | .010* |  |

Note: Est = unadjusted estimate. β = standardised regression coefficient. SE = standard error. 95% CI = 95% confidence interval. SES = socioeconomic status, including educational attainment, occupation, and household income. Standard errors were clustered at the family level to account for the non-independence of observations (i.e., two twins per family). **p*<.05 ****p*<.001.

## **Deviations from the pre-registration**

There was a minor deviation from the pre-registration of our analyses. We included an additional analysis, to test whether children born on Sunday had more favourable outcomes across all outcome measures in terms of a cumulative score. For this, a composite score was created across all outcome measures, after reverse-coding the emotionality composite. All other analyses were true to the pre-registration.
